# Two Cases of Primary Tuberculosis of the Prostate Reported to the Louisville Clinical Society*Stenographically reported for this journal by C. C. Mapes, Louisville, Ky.

**Published:** 1898-06

**Authors:** William C. Dugan

**Affiliations:** Professor of the Principles and Practice of Surgery and Surgical Pathology in the Louisville Medical College, etc., Louisville, Ky.


### TWO CASES OF PRIMARY TUBERCULOSIS OF THE PROSTATE REPORTED TO THE LOUISVILLE CLINICAL SOCIETY.*

By WILLIAM C. DUGAN, M.D.

Professor of the Principles and Practice of Surgery and Surgical Pathology in the Louisville Medical College, etc., Louisville, Ky.

I have here two gentlemen, brothers, private patients, who have kindly consented to come before you for examination, and who present a very unusual clinical feature. They are from Kentucky, and they have been suffering for a number of years with, first, irritation of the bladder, being unable to retain their urine more than half an hour at a time; symptoms of stone in the bladder, the passage of blood, pus, etc. They are unable to sleep at night, one of them especially having to be up nearly all the time emptying his bladder, and this patient told me, coming here to-night, that he was unable to sleep at all the first part of the night, that he was relieving his bladder almost continually. Pain is very great in both cases.

Some time ago the older of the two gentlemen came here to consult me for disease of his left testicle, which was then in an ulcerating state, and upon examination I found it to be in an advanced stage of tuberculosis, both the testicle and epididymis being involved. I also found that the cord was involved as high up as I could reach and almost as large as my finger. Pain at that time, owing to involvement of the skin and the immense amount of inflammation, was very great, and immediate operation was advised. The opposite testicle was also involved, but not nearly so far advanced. There were no adhesions, the epididymis was large, hard and painless. I simply removed the left testicle, leaving the right intact, as it appeared to be only slightly diseased. I did not examine his prostate at that time, expecting to do so before he returned home, but he went home while I was sick and the matter was neglected. The wound healed fairly well. I should have said upon examining the specimen I found that the cord—while I took it out as far up in the cavity as I possibly could, opening the inguinal canal, making the wound as large as I could, then with retractors went down as deeply into the pelvis as possible, lifting the cord out of the wound, hoping by this means to get beyond the disease, but I failed to do so. I left remnants of the disease in the stump. When the testicle was laid open there was very little of the parenchyma left and there were tubercular deposits throughout between the testicular structures. The epididymis was destroyed, and the cord presented one of the most interesting pathological conditions that I have ever met with. The tubercular deposits looked much like beans with the hull laid open, the cord being filled

*Stenographically reported for this journal by C. C. Mapes, Louisville, Ky.

with such deposits from one end to the other. Upon section with the knife it made a grating sound, the tubercules having undergone caseation.

He returned home and has had, just as we would expect, more or less discharge from the proximal end of the cord because of the fact that I did not get beyond the limit of the disease.

He came back to the city this morning and I examined him and asked him to come here to-night.

In the younger subject there is no involvement of the epididymis, but he gives a history of stone in the bladder. When the searcher was introduced we found his bladder very much contracted, thus making the examination very unsatisfactory. When examined per rectum we found that characteristic nodular feeling that you would expect in tuberculosis of the prostate and visicular seminales.

The older brother was then examined per rectum (the one whose left testicle had been removed), and I found a condition identical with the other one. I never in my life found two pathological conditions as much alike as in these two cases.

Now, the question I desire to ask is, what can be done for these two men? You will see that they are apparently in good health, generally speaking; they are anxious to be relieved of this distressing local condition and are willing to undergo almost anything to attain that end.

The question of castration has been considered. Will this be of as much advantage in tuberculosis of the prostate with testicular involvement as for enlargement of the prostate in old men? The first case operated upon in this city for bilateral tubercular testis where there was prostatic enlargement was performed in 1887. The man had just such a condition as you observe in the younger of the two men before you; the testicles were removed, and I told him the next operation would be removal of the prostate gland, but to my great surprise his symptoms from that time on improved, and in the course of three or four months instead of urinating every fifteen or twenty minutes he could retain his urine for an hour or more; he slept nearly all night and experienced very little of the paroxysmal pain which had been so common up to that time. He went on and entirely recovered from tuberculous prostatitis. I examined him not long ago and the prostate has become a hard mass not more than half as large as the phalanx of the finger. Now, the question is, can we expect such a result by double castration in the younger case before us, or shall we remove the tuberculous prostate and leave the testicles intact? If we leave the prostate as it is we are sure to have involvement of both testicles sooner or later. In case there appears to be no involvement of either testicle at present, but I am satisfied if proper treatment is not promptly instituted the result will be the same as in the other case where I removed the left testicle some time ago. I am almost certain in the first case that the prostate was the seat of the primary tuberculosis, and that the testicle was affected secondarily.

I would like to hear from some of the members of the society in regard to the advisability of double castration in the second case, or whether it is best to remove the prostate.

There is no other complication in either case; there is no history or evidence of tuberculosis elsewhere in the body. I remember several years ago having presented before the old Louisville Medical Society, now extinct, a specimen of tubercular testis, and in presenting the specimen certain members of the society took the ground that such operations should not be performed, inasmuch as this deposit only indicated a a general tuberculosis, not being willing to admit a local tuberculosis, which all of you will join me in saying is very common at the present time.

There is no history of specific or venereal disease in either case; both of the men are farmers and have lived in the country all their lives. The older one is forty, the younger thirty-eight years of age.

DISCUSSION.

DR. CARL WEIDNER: I do not desire to say anything about Dr. Dugan's cases from a surgical standpoint, but will leave that for some of the surgeons present. It is rather remarkable that both these patients should be affected with tuberculosis in identically the same locality; and further, that neither shows evidence of the disease elsewhere. However, that local tuberculosis occurs frequently, as stated by the reporter, I am prepared to admit. Still I look upon these cases as curiosities, being identical, with a family history that is practically without fault.

I do not believe very much in removing the testicles for the cure of prostatic enlargement in general. although I am aware the surgeons think otherwise, never having been able to convince myself of its utility. In a condition of tuberculosis particularly, I am doubtful whether it would be of any benefit. If the prostate is filled with caseous material, which I venture to say is a fact, I see no possibility of lessening the amount or of deriving benefit in cicatrization, condensation or fibrosis, the natural processes of cure we might say in tuberculous affections elsewhere, by removal of the testicles. I can see no reason for carrying out this procedure. If an operation can be done which shall have for its object the removal of the affected structures, then I believe it should be performed. Of course, in the one brother with the diseased testes and epididymis, these structures should be removed.

DR. T. P. SATTERWHITE: There is unquestionably an enlargement of the prostate in both cases. The vesicules seminales are distinctly involved in one case, and there seems also to be a deposit in the other, probably of the same character of material. Both these conditions will cause irritation of the bladder. It has been conclusively proven by operation, in one of the cases at least, that the trouble is tuberculous, and my opinion is that all tuberculous deposits, no matter where located, should be removed. The only suggestion I could offer would be to make an opening above the symphisis pubis, distending the bladder as far as possible, and make an examination of the interior of the bladder. My reason for suggesting this is that, if an operation should be performed and there should be fungous growths or tuberculous deposits within the bladder, it would be extremely unfortunate while performing the operation to omit the removal of any diseased structure. It would be well to investigate the interior of the bladder by ocular as well as digital means. I do not know what the result of the latest investigation is in regard to the transmission of tuberculosis to the ovum in cases of this kind, and this feature would be interesting for us to consider, and if it can be communicated in this way it would another strong argument in favor of operative interference—if there is the least danger of transmitting the disease to the offspring.

DR. W. C. DUGAN: Answering Dr. Satterwhite: According to all modern authorities these men are sterile from the earliest deposit of tuberculous material in the epididymis. This is perfectly natural and reasonable when the location of the deposit is considered, viz.: in the wall of the excretory ducts, practically filling the caliber of these ducts, so if the secreting portion of the testicle were normal, it is shut off mechanically from the prostate by virtue of the deposit in the epididymis. This is borne out by the statements of both these gentlemen. One of them has two children, the youngest twelve years old; the other has one child about six years old, and the oldest patient states he first began to have symptoms of bladder trouble, etc., twelve years ago, and in the other case it has been five years since he first noticed any symptoms.

In regard to operative treatment: My idea is to open up the perineum from below and explore it thoroughly and determine as near as I can what is to be done and then proceed to remove the tubercular deposit. I thought of opening from above, but the bladder is so contracted that I could hardly examine with an ordinary searcher for stone, so there is no room.

I should have stated that, on examination of the urine by the method of Ultzman, taking three bottles and securing three samples of the urine, one at the first washing, one from about the middle third, and then the last squeezing of the bladder, the first was clear, the second contained a little deposit, and the third contained quite a large amount of sediment. This shows conclusively that the the disease is prostatic, and as the bladder contracts the contents of the gland is squeezed out.

